# Design and Aerodynamic Analysis of a Flapping Mechanism for Foldable Biomimetic Aircraft

**DOI:** 10.3390/biomimetics10010061

**Published:** 2025-01-16

**Authors:** Shuai Yan, Yongjun Zhou, Shuxia Jiang, Hao Xue, Pengcheng Guo

**Affiliations:** 1 Department of Vehicle Engineering, School of Mechanical and Intelligent Manufacturing, Changsha 410004, China; yan15195962540@163.com (S.Y.); jiangshuxia_2004@126.com (S.J.); 20241200412@csuft.edu.cn (H.X.); 2 Automotive Parts Research Institute, Hunan University of Technology, Hengyang 421002, China; gpch860429@163.com

**Keywords:** bio-inspired design, kinematic simulation, aerodynamic performance, foldable wings, CFD simulation

## Abstract

This study investigates the unsteady aerodynamic mechanisms underlying the efficient flight of birds and proposes a biomimetic flapping-wing aircraft design utilizing a double-crank double-rocker mechanism. Building upon a detailed analysis of avian flight dynamics, a two-stage foldable flapping mechanism was developed, integrating an optimized double-crank double-rocker structure with a secondary linkage system. This design enables synchronized wing flapping and spanwise folding, significantly enhancing aerodynamic efficiency and dynamic performance. The system’s planar symmetric layout and high-ratio reduction gear configuration ensure movement synchronicity and stability while reducing mechanical wear and energy consumption. Through precise modeling, the motion trajectories of the inner and outer wing segments were derived, providing a robust mathematical foundation for motion control and optimization. Computational simulations based on trajectory equations successfully demonstrated the characteristic figure-eight wingtip motion. Using 3D simulations and CFD analysis, key parameters—including initial angle of attack, aspect ratio, flapping frequency, and flapping speed—were optimized. The results indicate that optimal aerodynamic performance is achieved at an initial angle of attack of 9°, an aspect ratio of 5.1, and a flapping frequency and speed of 4–5 Hz and 4–5 m/s, respectively. These findings underscore the potential of biomimetic flapping-wing aircraft in applications such as UAVs and military technology, providing a solid theoretical foundation for future advancements in this field.

## 1. Introduction

Birds achieve efficient flight through flapping, which generates high lift by utilizing unsteady aerodynamic effects [1,2,3]. Due to their exceptional flight capabilities, birds can navigate complex environments such as forests, swamps, and valleys with ease. Based on biomimetic principles, bird- and insect-inspired flapping-wing aircraft exhibit a high degree of biomimicry and excellent stealth performance. These aircraft demonstrate significant potential in military and drone applications, thereby making them a prominent focus of research worldwide [4,5,6,7,8]. Scholars have primarily investigated the aerodynamic characteristics and factors influencing efficient, stable flight in bionic flapping-wing aircraft. Liu (2016) explored the autonomous flight capabilities of MAVs at low Reynolds numbers, revealing the complex aerodynamic behaviors and efficient maneuverability when propulsion and control are generated through the wings [9]. Nakata (2011) combined CFD and wind tunnel experiments to analyze the unsteady aerodynamics of flexible flapping-wing MAVs, demonstrating improved flight control precision, especially in complex flow conditions [10]. Yeh and Hsu (2024) conducted experiments and simulations on a single-degree-of-freedom flapping mechanism mimicking rock pigeon wings, finding that PETG feathers outperformed PLA in lift and propulsion efficiency, showcasing their potential in FWMAV design [11]. Cai (2021) proposed a CFD data-driven aerodynamic model (CDAM) that accurately predicts flapping aerodynamics at various flight speeds, integrating quasi-steady and moving-body aerodynamics for enhanced flight control [12]. Sun (2021) modeled a seagull-like flapping-wing robot using a double-crank rocker mechanism, validating its design through ADAMS simulation [13]. Nguyen (2016) found that leading-edge reinforcement (LER) wings, mimicking insect wing flexibility, significantly improved the lift-to-drag and lift-to-weight ratios compared to rigid wings [14]. Yang (2018) studied the 3D flow field of a 20 cm span flapping wing, revealing key lift characteristics at varying wind speeds and frequencies, contributing to the understanding of flapping motion and MAV aerodynamics [15]. Akhter (2022) proposed an aerodynamic analysis method for biomimetic flaps, showing that spanwise deforming flaps outperform traditional designs in lift, drag reduction, and glide ratio, while delaying flow separation and suppressing turbulence [16]. In this context, this study initially analyzes the fundamental parameters and motion mechanisms of birds, subsequently designing a “flapping-folding” wing mechanism based on a double-crank-double-rocker structure, followed by kinematic analysis and the development of a complete 3D folding wing model. The primary innovation is founded upon the traditional crank-rocker mechanism, where an integrated virtual constraint approach is applied to the external linkage connection components. This approach not only achieves a more compact structural design but also improves the periodic consistency of the motion transmission function. Using the motion equations of the inner and outer wing segments, simulations were performed to simulate the realistic figure-eight trajectory of bird wingtips. Finally, using a 3D aerodynamic simulation platform, CFD software (ANSYS 2022R1) was used to simulate the folding-wing-flapping machine under low Reynolds number unsteady flow to analyze the effects of initial angle of attack, aspect ratio, flapping frequency, and velocity on aerodynamic characteristics. The results indicate that proper mechanism design and parameter optimization can markedly improve aerodynamic efficiency and flight stability.

## 2. Kinematic Analysis and Aerodynamic Simulation Methods

### 2.1. Mechanism of Bird Flight Movement

**Figure 1:**
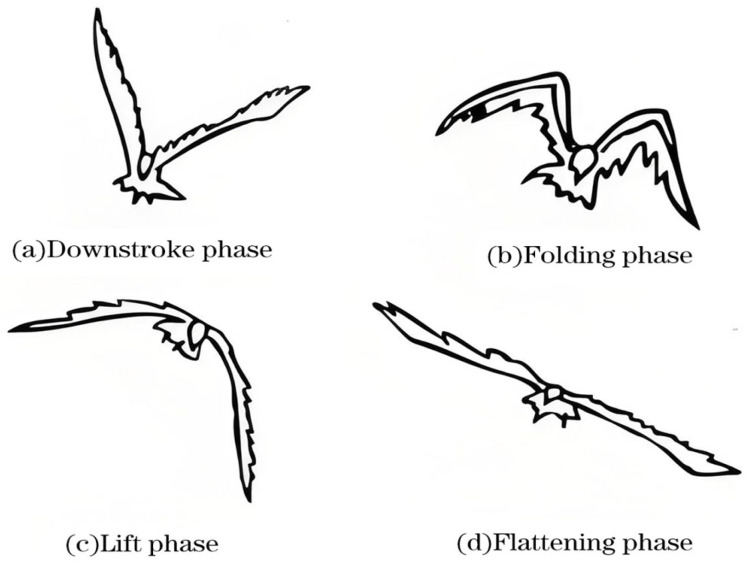
Sequential postures of bird wing flapping.

Birds modify their wing shape during flight to minimize aerodynamic drag and optimize energy efficiency. Figure 1 illustrates the four stages of the wingbeat cycle: downstroke, folding, upstroke, and flattening [17,18,19]. In the downstroke stage, the wings beat downward and twist forward, creating a negative angle of attack that generates both lift and thrust. During the folding stage, the outer wing briefly folds into a triangular shape due to inertia in preparation for the upstroke. In the upstroke stage, the scapula lifts first, the wrist slightly flexes backward, and the outer wing forms a drooped arch. During the flattening stage, the scapula descends from the peak of the upstroke, and the wings gradually flatten in preparation for the next wingbeat cycle.

**Figure 2:**
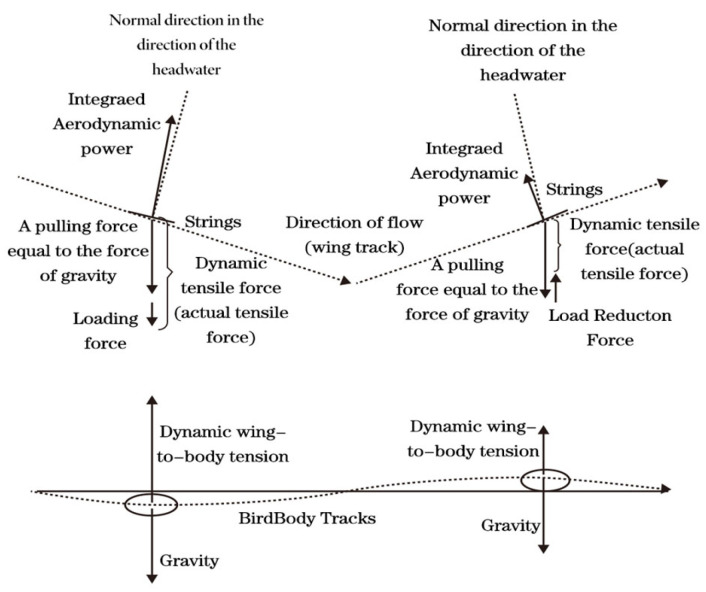
Analysis of sequential movement phases.

Birds optimize flight efficiency and aerodynamics throughout the wingbeat cycle by employing flexible wing deformation and active folding to adjust spanwise camber (see Figure 2). During the phase-wise motion process, birds generate forward thrust by exploiting dynamic variations in wing load. During the descending flight phase, the vertical lifting force between the main load and the wing increases dynamic lift in addition to the gravitational force of the main load, continuously applying load to the wings. (The main load maintains an upward acceleration, and the wings correspondingly increase the angle of attack and lift to stabilize linear flight). This loading force can be represented as the vector components along the direction of the wings’ forward motion. In the upward sliding flight phase, the vertical lifting force between the main load and the wing reduces dynamic lift from the main load’s gravitational force, continuously unloading the wings. (The main load maintains a downward acceleration, and the wings reduce the angle of attack or wing area, decreasing lift to sustain linear motion). This unloading force is also equivalent to a positive component along the direction of the wing’s motion, resulting in wave-like forward motion throughout the entire process.

Table 1 shows that the foldable wing designs exhibit significant advantages in bio-inspired aerial vehicles, particularly in performance optimization and mission adaptability, surpassing traditional fixed-wing designs. This innovative concept draws inspiration from the natural movements of birds and insects, with its advantages primarily manifesting in the following aspects:

### 2.2. Flapping Wing Mechanism Design

**Figure 3:**
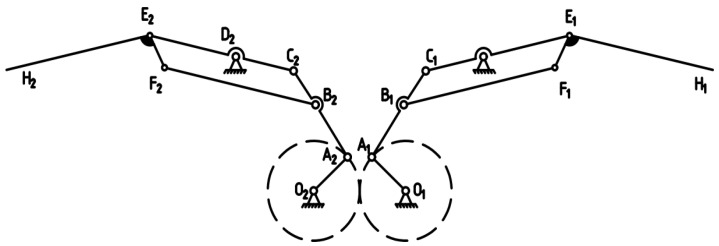
Schematic diagram of the flapping mechanism.

Research in bionics has shown that emulating the flapping–folding motion observed in bird wings can significantly improve the aerodynamic efficiency of flapping-wing aerial vehicles [20,21]. Building upon this insight, this paper presents a novel bionic flapping-wing aircraft mechanism that incorporates a two-stage foldable design, as depicted in Figure 3. This innovative mechanism refines the traditional double-crank double-rocker flapping mechanism by adding a two-rod, three-joint secondary linkage. This design allows for the synchronized control of wing flapping and extension folding, enhancing operational efficiency.

In the system, joints $O_{1}$ and $O_{2}$ serve as hinge points between the wing and frame, with a motor driving crank rotation $O_{1}A_{1}$, and a gear mechanism synchronously driving the other crank’s rotation $O_{2}A_{2}$. The linkages on both sides ($C_{1}E_{1}$ and $C_{2}E_{2}$) rotate about hinge points $D_{1}$ and $D_{2}$ to produce synchronized flapping motion in the inner wing segments. The quadrilateral mechanism ($C_{1}D_{1}F_{1}B_{1}$ and $C_{2}D_{2}F_{2}B_{2}$) effectively drives the movement of the outer wing segments via geometric deformation. Additionally, the relative rotation $C_{1}E_{1}$ and $C_{2}E_{2}$ between the components and the outer wings ($E_{1}H_{1}$ and $E_{2}H_{2}$) precisely enables the extension and folding action of the wings. This design utilizes an optimal combination of linkages and motion transfer mechanisms to ensure both the complexity and synchronization of bionic flapping, thus meeting the flapping and folding control requirements of the bionic aircraft. To verify the motion determinacy of the mechanism, a systematic evaluation is conducted using a degree of freedom analysis method. Based on the formula for calculating degrees of freedom:

(1) $$F=3n-2P_{L}-P_{H}$$

where n is the number of active components, $P_{L}$ is the number of lower pairs, and $P_{H}$ is the number of higher pairs. For this double-crank mechanism, the number of active components is n=3, the number of lower pairs is $P_{L}=4$, and the number of higher pairs is $P_{H}=0$, resulting in a calculated degree of freedom of F=1. Since the secondary linkage’s degree of freedom is zero, the entire system retains a degree of freedom of one, ensuring the precision and stability of the flapping mechanism’s movement. Drawing on the structural design from [22] this paper integrates the inner and outer wing rods during the hinging process to achieve a more compact structure. The meticulously designed double-crank–double-rocker mechanism ensures synchronized motion and consistent flapping amplitude across both wings, significantly boosting the aircraft’s stability. This synchronized design plays a crucial role in preventing instability due to uncoordinated wing movements, while its efficient structural layout further reduces mechanical wear and energy consumption.

The transmission system features a planar symmetrical layout and high-ratio reduction gears, effectively ensuring the synchronicity and stability of wing movements. Moreover, the system’s compact layout and optimized transmission efficiency provide robust mechanical support for the prolonged operation and practical application of the flapping-wing mechanism. This integration also aims to enhance the regularity of the H-point motion function, thereby facilitating innovative optimization. The results of these improvements are demonstrated in Figure 3.

### 2.3. Equations of Motion of Wing Structure

**Figure 4:**
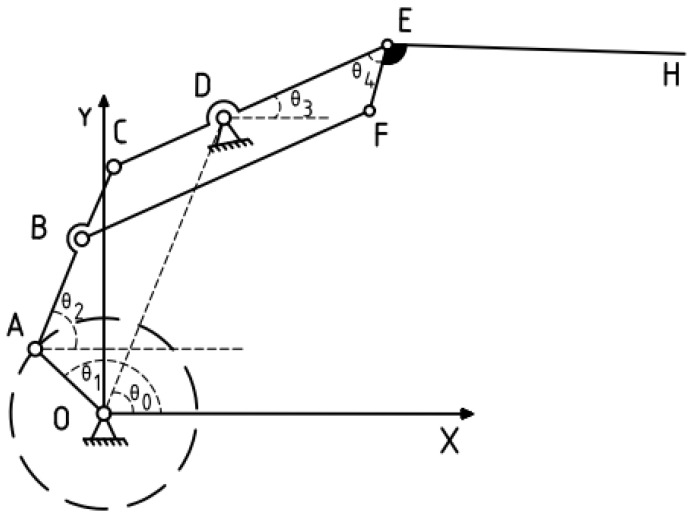
Schematic diagram of wing structure optimization.

The analysis of bird wing movement and the development of a wing model can replicate the “flapping folding” actions observed during bird flight. A simplified model was developed based on the wing movement pattern, as illustrated in Figure 4, with point O as the gear center, OA as the crank, and AC as the link rod of known length. Parallelogram BCEF represents the inner wing segment, with point D representing the fixed support point. EH represents the outer wing section, which is articulated with the inner segment by a hinge. The trajectory equations for the inner and outer wing segments are primarily represented by points E and H.

The *X*-axis is set as the horizontal ground. Given an angular velocity of $\omega_{1}$ rad/s, the crank OA angle determine at any moment:

(2) $$\theta_{1}=\omega_{1}t$$

where $\theta_{0}$ is the angle between the auxiliary line OD and the horizontal ground, as shown in Figure 3; $\theta_{3}$ is the angle of the inner wing section relative to the horizontal ground during motion; and $\theta_{4}$ is the folding angle of the outer wing relative to the inner wing.

Based on the motion of the inner wing section of the flapping-wing machine, the trajectory of point E is determined through geometric relations:

(3) $$\left\{\begin{array}{l} X_{E}=\left[L_{\mathrm{OA}}\cos\;(\theta_{1})+L_{\mathrm{AC}}\cos(\theta_{2})\right]+L_{\mathrm{CE}}\cos(\theta_{3})\\ {\;Y}_{E}=\left[L_{\mathrm{OA}}\sin\;(\theta_{1})+L_{\mathrm{AC}}\sin(\theta_{2})\right]+L_{\mathrm{CE}}\sin(\theta_{3}) \end{array}\right.$$

The coordinates of point E relative to point C can be expressed as:

(4) $$\left\{\begin{array}{l} X_{E}=X_{C}+L_{\mathrm{CE}}\cos(\theta_{3})\\ {\;Y}_{E}=Y_{C}+L_{\mathrm{CE}}\sin(\theta_{3}) \end{array}\right.$$

Let the length of the crank OA be $L_{\mathrm{OA}}$, the rotation angle be $\theta_{1}$, and the angular velocity be $\omega_{1}$. The coordinates of the end point A of the crank are:

(5) $$\left\{\begin{array}{l} X_{A}=L_{\mathrm{OA}}\cos(\theta_{1})\\ {\;Y}_{A}=L_{\mathrm{OA}}\sin(\theta_{1}) \end{array}\right.$$

Point C is connected to point A via the connecting rod AC, so the coordinates of point C depend on the coordinates of point A and the angle of the connecting rod AC, which is $\theta_{2}$. Specifically, the coordinates of point C are:

(6) $$\left\{\begin{array}{l} X_{C}=X_{A}+L_{\mathrm{AC}}\cos(\theta_{2})\\ {\;Y}_{C}=Y_{A}+L_{\mathrm{AC}}\sin(\theta_{2}) \end{array}\right.$$

Substituting the coordinates of point A, the coordinates of point C are:

(7) $$\left\{\begin{array}{l} X_{C}=L_{\mathrm{OA}}\cos(\theta_{1})+L_{\mathrm{AC}}\cos(\theta_{2})\\ {\;Y}_{C}=L_{\mathrm{OA}}\sin(\theta_{1})+L_{\mathrm{AC}}\sin(\theta_{2}) \end{array}\right.$$

The derivation of $\theta_{2}$ can be based on the following formula:

(8) $$L^{2}oc={L^{2}}_{OA}+{L^{2}}_{AC}-2L_{OA}\cdot L_{OA}\cdot \cos\;(\theta_{2}-\theta_{1})$$

The derived angle $\theta_{2}$ is:

(9) $$\theta_{2}=\theta_{1}+\cos\left(\frac{{L^{2}}_{OA}+{L^{2}}_{AC}-{\mathrm{OC}}^{2}}{2\cdot L_{\mathrm{OA}}\cdot L_{\mathrm{AC}}}\right)$$

By substituting Equations (9) and (10) into Equation (8), the coordinates of point C are derived as:

(10) $$\left\{\begin{array}{l} X_{C}=L_{\mathrm{OA}}\cos\;(\theta_{1})+L_{\mathrm{AC}}\cos\;(\theta_{1}+\cos^{-1}(\frac{{L^{2}}_{OA}+{L^{2}}_{AC}-{\mathrm{OC}}^{2}}{2\cdot L_{\mathrm{OA}}\cdot L_{\mathrm{OA}}}))\\ {\;Y}_{C}=L_{\mathrm{OA}}\sin\;(\theta_{1})+L_{\mathrm{AC}}sin(\theta_{1}+\cos^{-1}(\frac{{L^{2}}_{OA}+{L^{2}}_{AC}-{\mathrm{OC}}^{2}}{2\cdot L_{\mathrm{OA}}\cdot L_{\mathrm{OA}}})) \end{array}\right.$$

Point D is a fixed structural point, and the angles of points C and D relative to the *X*-axis are equal. Using the coordinates of point C, the coordinates of point D can be derived as:

(11) $$\left\{\begin{array}{l} X_{D}=\left[L_{OA}\cos\left(\theta_{1}\right)+L_{AC}\cos\left(\theta_{1}+\cos^{-1}(\frac{{L^{2}}_{OA}+{L^{2}}_{AC}-OC^{2}}{2\cdot L_{OA}\cdot L_{OA}})\right)\right]+L_{3}\cos(\theta_{3})\\ Y_{D}=\left[L_{OA}\sin\left(\theta_{1}\right)+L_{AC}\sin\left(\theta_{1}+\cos^{-1}(\frac{{L^{2}}_{OA}+{L^{2}}_{AC}-OC^{2}}{2\cdot L_{OA}\cdot L_{OA}})\right)\right]+L_{3}\cos(\theta_{3}) \end{array}\right.$$

Using the arctangent function with Equations (9) and (10), $\theta_{3}$ is:

(12) $$\theta_{3}=\arctan\left(\frac{L_{OD}\sin(\theta_{0})-L_{OA}\sin(\theta_{1})}{L_{CD}\cos(\theta_{0})-L_{OA}\cos(\theta_{1})}\right)$$

By combining Equations (2) through (12), the specific trajectory of point E can be determined:

(13) $$\left\{\begin{array}{l} X_{E}=\left[L_{\mathrm{OA}}\cos(\theta_{1})+L_{\mathrm{AC}}\cos(\theta_{1}+\cos^{-1}(\frac{{L^{2}}_{OA}+{L^{2}}_{AC}-OC^{2}}{2\cdot L_{\mathrm{OA}}\cdot L_{\mathrm{AC}}}))\right]+L_{\mathrm{CE}}\cos(arctan(\frac{L_{\mathrm{OD}}\sin(\theta_{0})-L_{\mathrm{OA}}\sin\;(\theta_{1})}{L_{\mathrm{CD}}\cos(\theta_{0})-L_{\mathrm{OA}}\cos(\theta_{1})}))\\ X_{E}=\left[L_{\mathrm{OA}}\sin(\theta_{1})+L_{\mathrm{AC}}sin(\theta_{1}+\cos^{-1}(\frac{{L^{2}}_{OA}+{L^{2}}_{AC}-OC^{2}}{2\cdot L_{\mathrm{OA}}\cdot L_{\mathrm{AC}}}))\right]+L_{\mathrm{CE}}sin(arctan(\frac{L_{\mathrm{OD}}\sin(\theta_{0})-L_{\mathrm{OA}}\sin\;(\theta_{1})}{L_{\mathrm{CD}}\cos(\theta_{0})-L_{\mathrm{OA}}\cos(\theta_{1})})) \end{array}\right.$$

Furthermore, based on the properties of parallelogram BCE, in triangle △CBE, ∠CBF can be derived using the cosine rule:

(14) $$\theta_{4}=∠\mathrm{CBF}=\cos^{-1}(\frac{{L^{2}}_{CB}+{L^{2}}_{BF}-{L^{2}}_{CF}}{2\cdot L_{CB}\cdot L_{BF}})$$

Since EFH is a fixed structure and ∠FEH is a constant, the coordinates of point H are derived as:

(15) $$\left\{\begin{array}{l} X_{H}=X_{E}+L_{EH}\cos(\theta_{4}+∠FEH)\\ Y_{H}=Y_{E}+L_{EH}\sin(\theta_{4}+∠FEH) \end{array}\right.$$

### 2.4. Flapping Wing Aircraft Modeling

**Figure 5:**
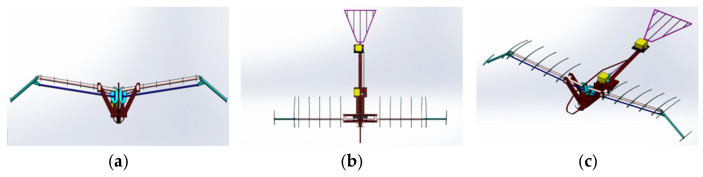
Skeleton model schematic. (**a**) Front view; (**b**) Top view; (**c**) Isometric view.

By simplifying the motion characteristics of bird wings, this skeletal model retains essential movement characteristics while maintaining aerodynamic and structural integrity during flight (see Figure 5). The design and modeling were carried out on the Solidworks platform, resulting in a comprehensive skeletal framework that includes the main body, tail, inner wing section, and outer wing section, as shown in Table 2.

### 2.5. Motion Simulation of Flapping Wing Aircraft

**Figure 6:**
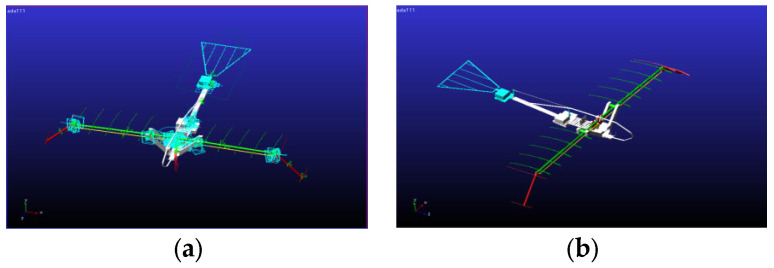
Motion simulation effects schematic. (**a**) Motion simulation with constraints; (**b**) Motion simulation with loading.

Based on the derived equations of motion for both the inner and outer wing sections, a kinematic analysis was conducted of the designed foldable-wing-flapping aircraft. The flapping mechanism was optimized according to the specified design parameters and predefined mechanical constraints [23]. Motion simulations and analyses were performed using the ADAMS simulation platform. The resulting motion trajectories of the inner and outer wing sections are shown in Figure 6.

### 2.6. Construction of 3D Pneumatic Simulation Platform

This study investigates the effects of the initial wing angle of attack, aspect ratio, flapping frequency, and flapping speed on aerodynamic characteristics under dynamic conditions through multiple sets of 3D aerodynamic simulations and data analyses. A total of 25 initial angles of attack, 11 aspect ratios, 13 flapping frequencies, and 5 speed settings were considered in the simulation conditions. The initial angle of attack varied from −12° to 12°, the aspect ratio from 4.5 to 5.5, the flapping frequency from 2 Hz to 8 Hz, and the speed from 2 to 6 units. The aerodynamic characteristics of the foldable-wing aircraft were modeled using the virtual wind tunnel mode in XFlow software (ANSYS 2022 R1). The basic configuration parameters of the virtual wind tunnel are presented in Table 3.

**Figure 7:**
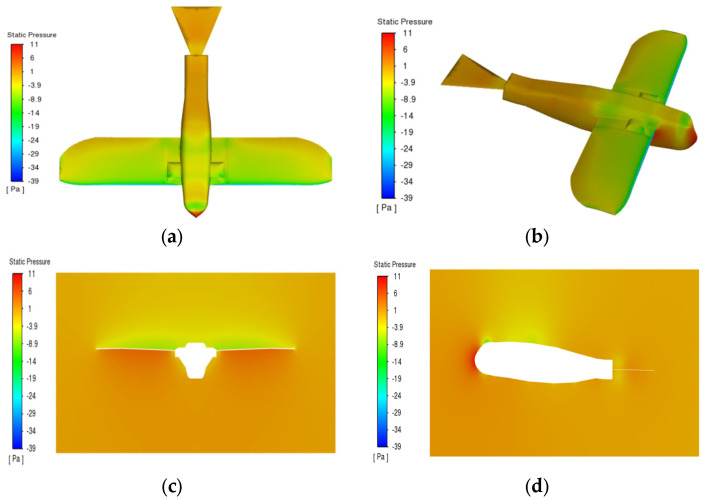
Pressure distribution contour plot of the wing in various directions of the model. (**a**) Front view; (**b**) Oblique side view; (**c**) Front view; (**d**) Side view.

This study employs the NACA 23,112 airfoil as the computational model to examine the effect of the initial angle of attack on aerodynamic characteristics, as shown in Figure 7 [22]. Simulations demonstrate how different factors under controlled conditions influence the bionic flapping-wing aircraft in ideal scenarios, with a primary focus on comparing lift and thrust coefficients under various conditions [27,28].

## 3. Analysis and Numerical Results

### 3.1. Kinematic Simulation Results of the Flapping Wing Mechanism

#### 3.1.1. Angular Displacement of Inner Wing Mechanism over Time

**Figure 8:**
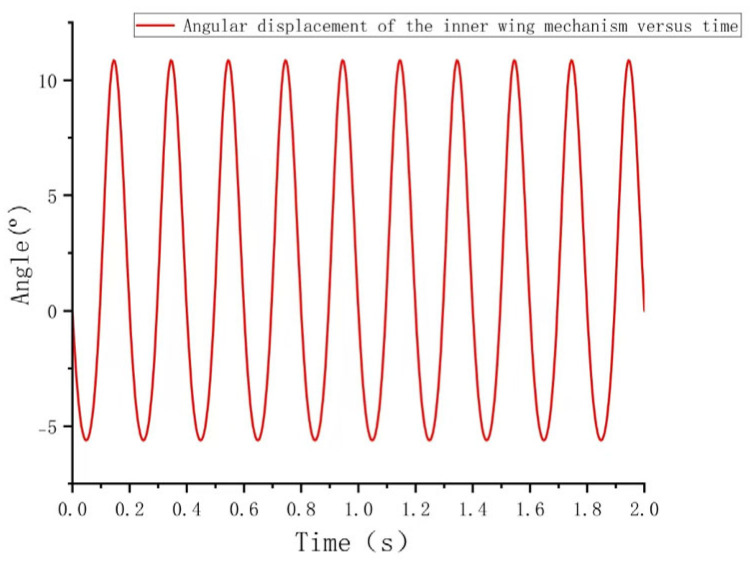
Angular displacement of the inner wing mechanism versus time.

As shown in Figure 8, the angular displacement of the inner wing mechanism displays distinct, periodic sinusoidal oscillations, with a period of approximately 0.2 s. During each cycle, the angular displacement increases from a negative value (approximately −10 degrees) to a positive peak (around 10 degrees), and then decreases to a negative peak, demonstrating the characteristic reciprocating motion. Over time, the angle alternates consistently between positive and negative values, indicating that the inner wing mechanism’s motion is both symmetrical and highly repeatable.

#### 3.1.2. Displacement of the Outer Wing Mechanism

**Figure 9:**
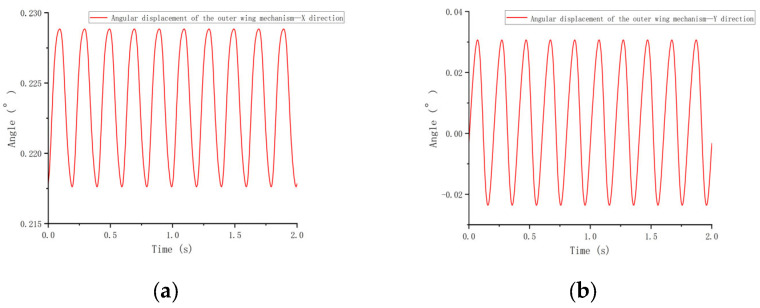
Angular Displacement of the Outer Wing Mechanism (X, Y Directions). (**a**) X direction; (**b**) Y direction.

Figure 9 illustrates that the displacement of the outer wing mechanism (center of mass) in both the X and Y directions exhibits periodic, sine wave-like fluctuations. Figure 8 shows that the displacement in the X direction gradually increases from a negative value, peaks at a positive value, and then returns to negative, indicating the outer wing’s reciprocating motion along the *X*-axis. The peak displacement in each cycle gradually diminishes over time, revealing a damping effect, likely caused by energy losses due to air resistance or mechanical friction. In contrast, the *Y*-axis displacement shows smaller fluctuations with lower amplitude compared to the *X*-axis, suggesting that the vertical motion of the outer wing is constrained, with more energy focused along the *X*-axis. Overall, motion in the X direction is more prominent, marked by larger positive and negative oscillations, whereas the Y direction remains relatively stable.

#### 3.1.3. Torsion Angle at the Outer Wingtip Versus Time

**Figure 10:**
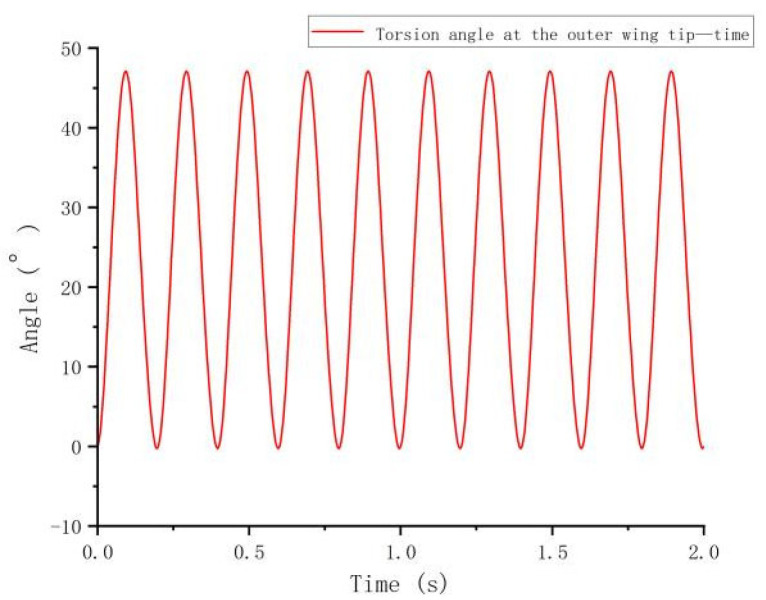
Torsion angle at the outer wingtip versus time.

Figure 10 illustrates the periodic oscillations of the torsional angle at the outer wingtip over time, fluctuating between increasing and decreasing phases. This pattern indicates that the wingtip reacts to periodic aerodynamic forces, such as lift and drag, during flight. Initially, the torsional angle increases sharply before settling into a stable oscillatory pattern. The analysis reveals regular variations in the maximum and minimum values of the torsional angle, illustrating dynamic adaptations under aerodynamic loading. Over time, the torsional angle consistently recovers and stabilizes, reflecting the system’s rhythmic adaptation to aerodynamic forces.

#### 3.1.4. Trajectory Diagram of Wing Movement

**Figure 11:**
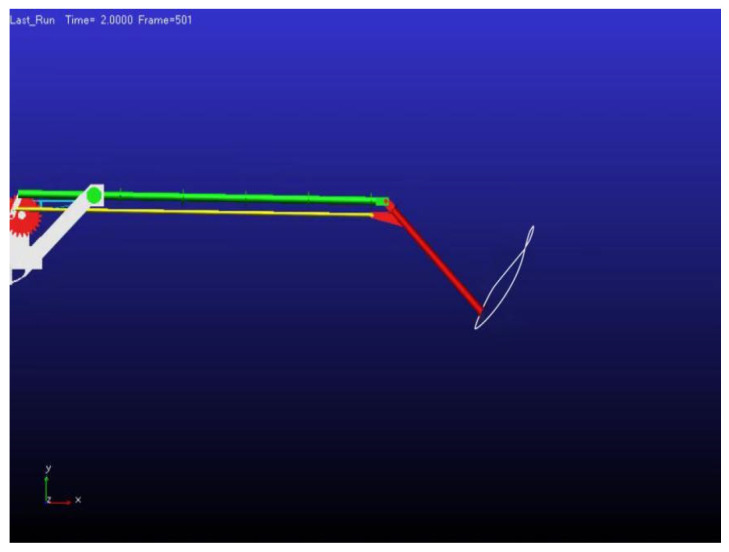
Trajectory diagram of wing movement.

After importing the model into the software and configuring all necessary constraints, the simulation is executed to replicate the up-and-down flapping of the inner wing section and the folding motion of the outer wing section. The motion trajectories for the entire cycle are subsequently derived and depicted in Figure 11. The simulation reveals that while the inner wing section performs a relatively simple up-and-down motion, the outer wing section undergoes a complex folding motion. Together, these movements produce a figure-eight motion pattern, closely mimicking the natural flapping behavior of birds during flight. The simulation analysis confirms that the designed flapping mechanism successfully replicates the desired motion, demonstrating that the biomimetic flapping-wing model exhibits high-quality kinematic performance and effectively simulates the flapping behavior of bird wings [29].

### 3.2. Effect of Initial Attack Angle of Flapping Wing on Aerodynamic Characteristics

The experimental conditions included an initial angle of attack ranging from −12° to 12°, with a step size of 1°, an inflow speed of 4 m/s, and a Reynolds number of $1.5\times 10^{5}$.

**Figure 12:**
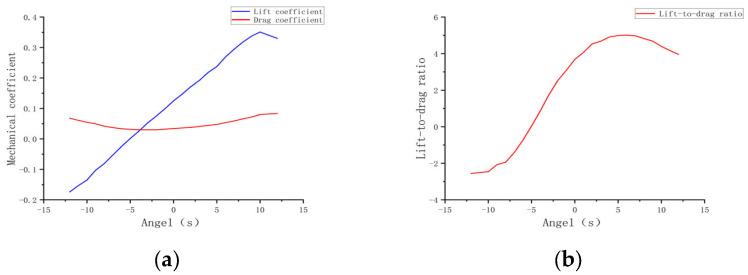
Curves at different initial angles attack. (**a**) Lift coefficient and drag coefficient curves; (**b**) Lift-to-drag ratio curve.

Figure 12a illustrates that as the angle of attack increases from −12° to 0°, the lift coefficient Cl gradually shifts from negative to positive, signifying a transition from negative to positive lift.

This suggests that at negative angles of attack, the wing fails to generate effective lift, but at near-zero angles of attack, it begins to operate efficiently, producing positive lift, making it suitable for takeoff and climb phases. As the angle of attack continues to increase to 12°, the lift coefficient peaks and then slightly declines, suggesting that beyond a certain critical angle, the rate of lift increase slows down. The drag coefficient generally shows an upward trend as the angle of attack increases from −12° to 12°, particularly in the positive angle range. Initially, the drag coefficient Cd is low at negative angles of attack, but it peaks as the angle of attack increases.

This suggests that as the angle of attack increases, air resistance rises, likely due to airflow separation and vortex formation. Figure 12b shows that as the angle of attack increases, the lift-to-drag ratio L/D significantly improves between 0° and 9°, reaching a maximum value of 4.6833 at 9°. At 0°, the lift-to-drag ratio L/D is 3.6873, and as the angle of attack increases to 9°, the ratio progressively rises, indicating strong aerodynamic performance. However, when the angle of attack surpasses 9°, the lift-to-drag ratio L/D begins to decrease, suggesting that the lift increase can no longer offset the drag increase, signaling a drop in aerodynamic efficiency [30].

**Figure 13:**
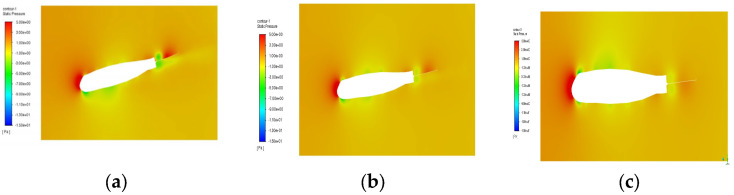

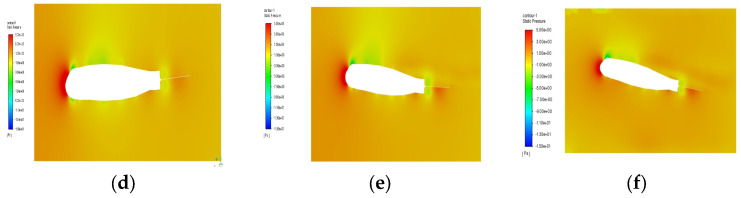
Pressure distribution contour plot of the wing at different initial angles of attack. (**a**) −12°; (**b**) −6°; (**c**) −1°; (**d**) 1°; (**e**) 6°; (**f**) 12°.

As shown in Figure 13, the initial angle of attack significantly influences the aerodynamic characteristics of the flapping-wing aircraft. Within the angle of attack range from 0° to 6°, the lift steadily increases, and the lift-to-drag ratio reaches its maximum, indicating optimal aerodynamic performance. However, when the angle of attack exceeds 6°, despite the continued increase in lift, the rapid rise in drag results in a decrease in the lift-to-drag ratio, potentially increasing the risk of the aircraft stalling [31].

### 3.3. Effect of Wing Aspect Ratio on Aerodynamic Characteristics of Flapping-Wing Aircraft

Constraints: The inflow velocity is set at 5 m/s, the wing-flapping angle θ is 60°, and the wing-flapping frequency is 4 Hz, resulting in a motion cycle of T = 0.25 s, with an initial angle of attack of 5°. The wing-flapping motion follows a quick-return characteristic, meaning that during one motion cycle, the downward flapping time exceeds the upward time, following a cosine variation pattern. A piecewise function is configured in XFlow to introduce the time difference. The expression for the variation in the flapping angle of the wing during one motion cycle of the flapping-wing aircraft is as follows [32]:

(16) $$\theta \;(t)=\left\{\begin{array}{ll} \alpha \cos(\frac{2\pi t}{T\;}+\phi_{1}) & 0\leq t\leq \frac{T}{2}\\ \alpha \cos(\frac{2\pi (t-\frac{T}{2})}{T\;}+\phi_{2}) & \frac{T}{2}\leq t\leq T \end{array}\right.$$

**Figure 14:**
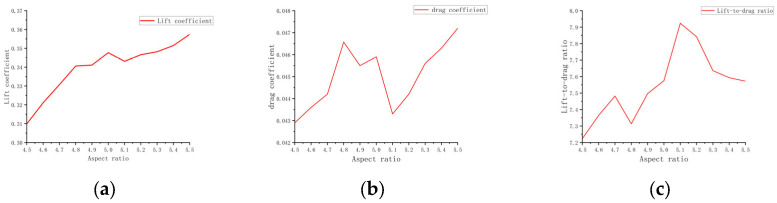
Curves of at different aspect ratios. (**a**) Lift coefficient; (**b**) Drag coefficient; (**c**) Lift-to-drag ratio.

Figure 14a shows a general upward trend in the lift coefficient as the aspect ratio increases. As the aspect ratio increases from 4.5 to 5.5, the lift gradually rises from 0.3099 to 0.3574. This suggests that a higher aspect ratio allows for more effective control of airflow over the wing, which enhances the efficiency of lift generation. Figure 14b shows that as the aspect ratio increases, the drag coefficient remains relatively stable, with only minimal variation. As the aspect ratio increases from 4.5 to 5.5, the drag coefficient rises from 0.0429 to 0.0472. Although there is a slight increase in drag, its increase is significantly smaller than that of the lift. This indicates that in the design of flapping-wing aircraft, increasing the aspect ratio does not lead to a significant increase in drag, and that the resulting increase in drag remains manageable. Overall, increasing the aspect ratio within a certain range is advantageous, as the increase in drag does not meaningfully offset the lift improvement. Figure 14c indicates that the lift-to-drag ratio exhibits an overall increasing trend as the aspect ratio increases. At an aspect ratio of 5.5, the lift-to-drag ratio is 7.572; when the aspect ratio is 5.1, the ratio peaks at 7.9238, after which minor fluctuations are observed. The lift-to-drag ratio reflects the aerodynamic efficiency of the aircraft. A higher ratio indicates that the aircraft produces more lift relative to the drag incurred. When the aspect ratio is between 5.1 and 5.5, the lift-to-drag ratio approaches its maximum, suggesting that this range offers the best aerodynamic efficiency, enabling the flapping-wing aircraft to maximize energy efficiency in flight.

**Figure 15:**
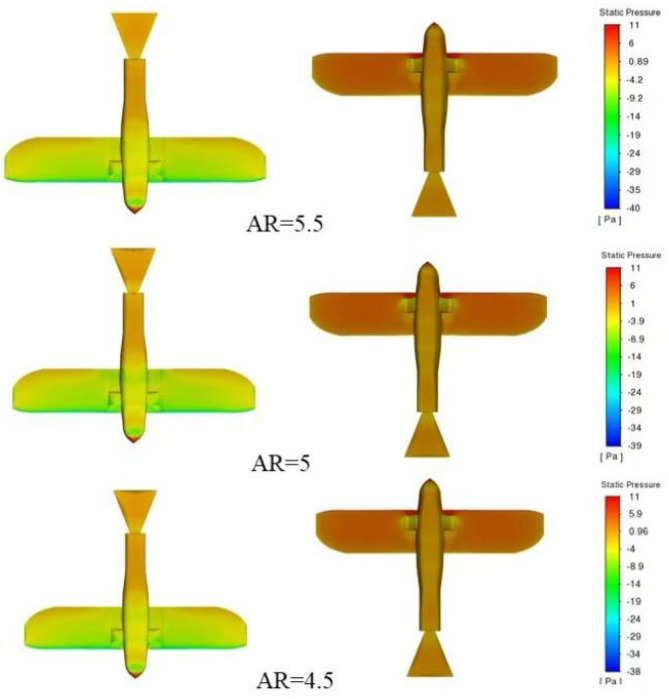
Pressure distribution contour plot of the wing at different aspect ratios.

Figure 15 shows that as the aspect ratio decreases from 5.5 to 4.5, the lift decreases accordingly, revealing a trend where lower aspect ratios result in a gradual reduction in lift. Correspondingly, the drag shows only minor variations. Although drag fluctuates slightly with changes in aspect ratio, the lift-to-drag ratio shows noticeable changes. Overall, the lift-to-drag ratio shows a positive correlation with the aspect ratio, meaning that an optimal range of aspect ratios leads to higher aerodynamic efficiency [33].

### 3.4. Effect of Flapping Frequency of Folded Wing on Aerodynamic Characteristics of Aircraft

**Figure 16:**
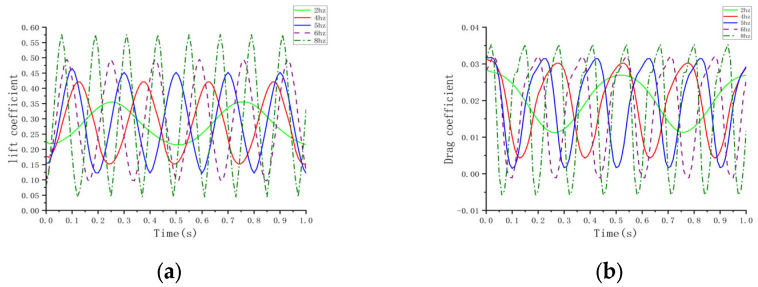
Curves at different frequencies. (**a**) Lift coefficient; (**b**) Drag coefficient.

**Figure 17:**
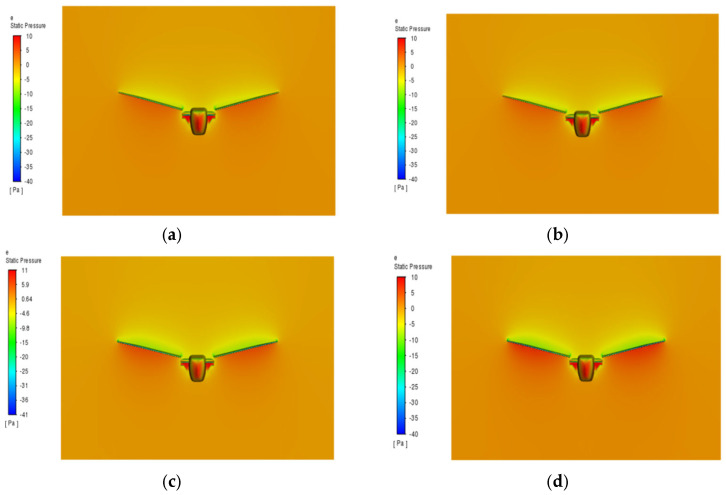
Pressure distribution contour plot of the wing at different frequencies. (**a**) 2 Hz; (**b**) 4 Hz; (**c**) 6 Hz; (**d**) 8 Hz.

Under experimental conditions involving an inflow velocity of 5 m/s, an initial wing angle of attack of 5°, and an aspect ratio of 3, simulations were conducted to analyze the lift coefficient, drag coefficient, and aerodynamic characteristics of the aircraft at different flapping frequencies. Figure 16a illustrates that, as the flapping frequency increases, the pressure difference between the wing’s upper and lower surfaces grows significantly, leading to increased fluctuations in the lift coefficient. In the low- to mid-frequency range (2 to 4–6 Hz), the lift coefficient increases steadily, with higher peaks and deeper troughs. At a higher frequency of 8 Hz, the lift coefficient exhibits more dramatic fluctuations, reflecting a pronounced aerodynamic response. Figure 16b demonstrates that the drag coefficient varies significantly with increasing frequency. In the 4–6 Hz range, the drag coefficient exhibits a negative peak, indicating a trend towards thrust generation. This suggests that the aircraft not only overcomes air resistance but also gains propulsion at this frequency, thereby enhancing efficiency. The data indicate that the drag coefficient remains relatively low at 4–5 Hz but increases sharply at 8 Hz. Based on the average lift and drag coefficients in Table 4, the lift-to-drag ratio reaches its peak in the 4–5 Hz range, indicating optimal aerodynamic efficiency. In this frequency range, the aircraft achieves significant lift while maintaining low drag, demonstrating excellent overall energy efficiency. Figure 17’s analysis of the airflow field reveals that, as the flapping frequency increases, the airflow speed around the wing increases significantly. During the downstroke, the airflow over the upper wing surface exceeds that over the lower surface, generating a significant pressure difference and contributing to lift generation. During the upstroke, increased airflow over the lower surface reverses the pressure difference, further enhancing lift. At the lowest point of the downstroke, the pressure difference reaches its maximum, aiding in lift generation; during the folded upstroke, airflow speed on the lower surface increases. The asymmetrical motion causes an uneven distribution of aerodynamic forces on the aircraft throughout the flapping cycle, resulting in greater net lift and improved aerodynamic efficiency. Overall, in the 4–5 Hz frequency range, the aircraft demonstrates optimal aerodynamic efficiency and balanced structural loading, making it suitable for efficient flight missions [34,35,36,37,38].

### 3.5. Effect of Flapping Speed of Folded Wing on Aerodynamic Characteristics of Aircraft

**Figure 18:**
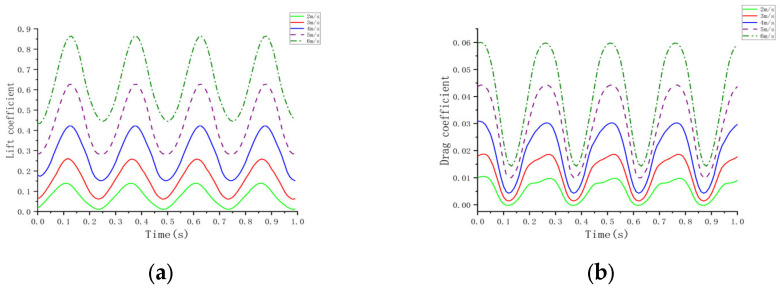
Curves of lift coefficient and drag coefficient at different speeds. (**a**) Lift coefficient; (**b**) D rag coefficient.

**Figure 19:**
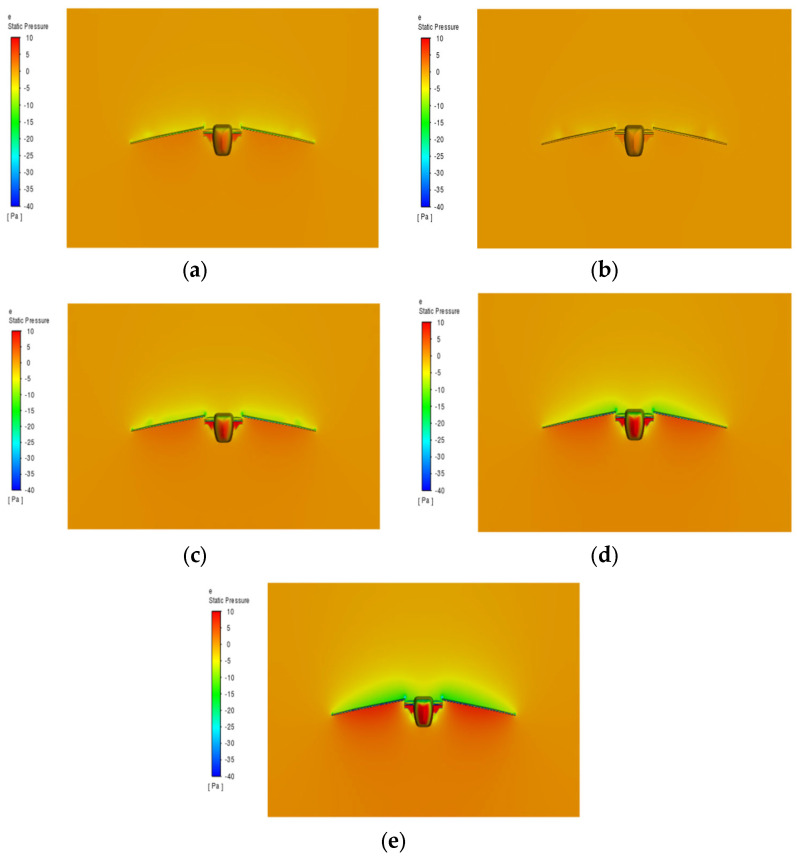
Pressure distribution contour plot of the wing at different frequencies. (**a**) 2 m/s; (**b**) 3 m/s; (**c**) 4 m/s; (**d**) 5 m/s; (**e**) 6 m/s.

Under experimental conditions with an inflow velocity of 5 m/s, an initial wing angle of attack of 5°, and an aspect ratio of 3, simulations were performed to analyze the effect of varying flapping speeds on the aerodynamic characteristics of the aircraft. Figure 18a illustrates that as the flapping speed increases, the pressure difference between the upper and lower surfaces of the wing increases significantly, resulting in substantial fluctuations in the lift coefficient. In the low to medium speed range (2–5 m/s), the lift coefficient increases steadily, with higher peak values and more pronounced troughs. At higher speeds (6 m/s), the lift coefficient fluctuates more drastically, indicating distinct aerodynamic responses. Figure 18b illustrates that the drag coefficient exhibits substantial variation as the flapping speed increases. In the medium speed range (4–5 m/s), the drag coefficient reaches a negative peak, indicating a positive thrust trend. This suggests that within this speed range, the aircraft not only overcomes air resistance but also generates thrust, thereby enhancing overall propulsion efficiency. Data analysis indicates that the drag coefficient is relatively low in the 4–5 m/s range but rises significantly at 6 m/s. The analysis of the average lift and drag coefficients in Table 5 reveals that the lift-to-drag ratio reaches its peak in the 4–5 m/s speed range, indicating optimal aerodynamic efficiency. Within this speed range, the aircraft generates substantial lift while maintaining low drag, thereby enhancing overall energy efficiency. At low speeds (2 m/s), despite the reduced lift, the low drag results in a high lift-to-drag ratio with minimal fluctuation, making it ideal for stable cruising conditions. At higher speeds (6 m/s and above), although lift increases, the significant rise in drag reduces the lift-to-drag ratio, making it more suitable for short, highly maneuverable flight operations. Figure 19 illustrates that as the flapping speed increases, the airflow speed around the wing increases significantly. During the downstroke, the airflow speed on the upper wing surface exceeds that on the lower surface, resulting in a considerable pressure difference that contributes to lift generation. During the upstroke, faster airflow on the lower surface inverts the pressure differential, further enhancing lift. The relative motion of the folding wing’s inner and outer segments during flapping induces complex variations in the airflow field. When the wing reaches its lowest point in the downstroke, the pressure differential peaks, facilitating lift generation. During the folded upstroke, airflow velocity along the lower surface increases. This asymmetrical motion causes an imbalance in the forces acting on the aircraft during each flapping cycle, leading to increased net lift and improved aerodynamic efficiency [39].

Overall, in the 4–5 m/s speed range, the aircraft demonstrates optimal aerodynamic efficiency and balanced structural loading, making it ideal for efficient flight missions. Lower speeds are better for stable cruising, whereas higher speeds are more appropriate for short-term high-maneuverability operations [40].

## 4. Discussion

As discussed in Section 3.1, the motion simulation of the foldable-wing flapping-wing vehicle resulted in the optimization of the motion trajectories for both the inner and outer wings. The simulation results indicate that the angular displacement of the inner wing exhibits periodic sinusoidal oscillations with a period of approximately 0.2 s and an amplitude ranging from −10° to 10°, demonstrating stable reciprocating motion. The centroid displacement of the outer wing in the X direction exhibits periodic oscillations, with the maximum displacement gradually decreasing, indicating a damping effect, while the displacement variation in the Y direction remains relatively small. The wingtip twist angle undergoes periodic oscillations due to aerodynamic loads. Furthermore, the motion trajectory of the outer wing approximates a figure-eight pattern, simulating the flapping motion observed in bird flight, thereby verifying the kinematic characteristics of this biomimetic flapping-wing mechanism.

Section 3.2, Section 3.3, Section 3.4 and Section 3.5 analyze the specific effects of the initial angle of attack, aspect ratio, flapping frequency, and flapping speed on the aerodynamic characteristics of the flapping-wing mechanism through experiments and simulations, aiming to quantify the optimization effects of these parameters on aerodynamic performance.

1. Initial Angle of Attack:
  Experimental results indicate that as the initial angle of attack increases from −12° to 12°, the lift coefficient transitions gradually from a negative to a positive value, with the maximum lift-to-drag ratio occurring at 9°, reaching 4.6833. At this point, the lift is maximized, and aerodynamic efficiency is optimal. As the angle of attack increases further, the drag coefficient rises substantially, resulting in a reduction in the lift-to-drag ratio, indicating a decline in aerodynamic efficiency beyond 9°.
2. Aspect Ratio:
  The increase in aspect ratio is positively correlated with the lift coefficient. When the aspect ratio increases from 4.5 to 5.5, the lift coefficient rises from 0.3099 to 0.3574, whereas the drag coefficient remains largely unchanged. The maximum lift-to-drag ratio of 7.9238 occurs at an aspect ratio of 5.1, resulting in optimal aerodynamic efficiency. Further increases in aspect ratio have minimal effect on drag, and thus aerodynamic performance remains optimal, within the range of 5.1 to 5.5.
3. Flapping Frequency:
  Variations in flapping frequency have a significant effect on both lift and drag coefficients. Within the frequency range of 4–5 Hz, the lift-to-drag ratio reaches its peak, resulting in optimal aerodynamic efficiency. As the frequency increases to 8 Hz, the fluctuations in lift and drag become more pronounced, resulting in a decline in aerodynamic efficiency. This suggests that the 4–5 Hz range is optimal for balancing aerodynamic performance, while higher frequencies are more suited for short-term high-maneuverability flight.
4. Flapping Speed:
  Simulation results indicate that, at a flapping speed of 4–5 m/s, the lift-to-drag ratio peaks at 6.4361, reflecting optimal aerodynamic efficiency. At lower speeds (2 m/s), both lift and drag remain relatively low, making this range suitable for stable cruising. At higher speeds (6 m/s), although lift increases, drag also rises substantially, making this range more suitable for short-term high-maneuverability missions.

By quantifying the effects of the initial angle of attack, aspect ratio, flapping frequency, and flapping speed on the flapping-wing mechanism, the study identifies an optimal combination of parameters within a defined range, significantly enhancing aerodynamic performance. These results provide valuable parameter references for the design of biomimetic flapping-wing aircraft.

## 5. Conclusions

This study, inspired by the flight mechanics of birds, proposes a “flapping folding” flapping wing mechanism and analyzes its structural motion. Initially, a 3D model of the folding wing was developed, and the motion trajectory equations for the inner and outer wing segments were derived. Simulations were conducted to analyze its motion characteristics. Subsequently, aerodynamic simulations using a 3D platform and CFD software were performed, yielding the following findings:

1. Aerodynamic forces and torsional oscillation of the outer wing: Periodic aerodynamic forces (i.e., lift and drag) cause the torsion angle at the outer wingtip to exhibit distinct periodic oscillations over time. Simulation results indicate that the torsion angle of the outer wing remains within a controlled range, suggesting strong stability and durability under complex aerodynamic conditions.
2. Figure-eight wing motion trajectory: The wing’s motion traces a figure-eight pattern, with the inner wing segment primarily responsible for the up-and-down flapping, while the outer segment folds during the motion. This trajectory mimics the agile flapping behavior of birds, ensuring continuous lift generation throughout flight, while folding reduces drag during the upstroke, thereby enhancing overall aerodynamic efficiency.
3. Aerodynamic performance optimization and flapping wing parameters: Simulation results indicate that optimal aerodynamic performance is achieved with an initial angle of attack of between 0° and 9°, with the lift-to-drag ratio peaking at 9°. When the angle of attack exceeds 9°, drag increases sharply, resulting in a significant decline in aerodynamic efficiency. Furthermore, with an aspect ratio of 5.1, the lift-to-drag ratio peaks, achieving optimal aerodynamic efficiency. A moderate increase in aspect ratio enhances lift with minimal drag increase, thereby improving overall aerodynamic efficiency. At a flapping frequency of 4–5 Hz, the lift-to-drag ratio is maximized, and aerodynamic efficiency is optimized, making it ideal for efficient flight tasks. Low frequencies (2 Hz) are suitable for stable cruising, while higher frequencies (6 Hz and above), though increasing lift, also significantly raise drag, making them more suited for short-term high-maneuverability missions. At a flapping speed of 4–5 m/s, the lift-to-drag ratio peaks, resulting in optimized aerodynamic efficiency. Lower speeds are ideal for cruising, while higher speeds are more suited for short bursts of high-maneuverability missions. Therefore, flapping-wing aircraft design should select the appropriate flapping speed based on the specific mission to optimize both aerodynamic performance and structural integrity.
4. Coordination between inner and outer wing segments and optimization suggestions: Simulations indicate that the inner and outer wing segments are well-coordinated during motion, though further optimization of their relative movement may be necessary in practical applications, depending on the specific flight mission. For instance, adjusting hinge stiffness or improving the crank-rod linkage could enhance energy efficiency and flight stability.

## 6. Patents

1. Yan Shuai et al., A Casual-Wear Bionic Flapping-Wing Aircraft (ZL 2023 2 2864225.9).
2. Yan Shuai et al., A Small Bionic Flapping-Wing Aircraft (ZL 2023 2 2864222.5).

## Figures and Tables

**Table 1:** Comparison between foldable wings and traditional fixed wings.

Characteristics	Foldable Wings	Traditional Fixed Wings
Flexibility	Features dynamic adjustment capability, allowing wing shape and span changes to adapt to mission requirements and flight environments, offering high adaptability.	Fixed structure without the ability to adjust wing shape or span, resulting in lower adaptability for complex or dynamic missions.
Aerodynamic performance	Can optimize lift-to-drag ratio through dynamic adjustment of wingspan and shape, significantly improving efficiency during takeoff, cruising, and descent.	Fixed wing structure limits aerodynamic optimization, making it less adaptable to varying flight phases.
Wind resistance	Capable of reducing frontal area by retracting wings, maintaining stability and control in high-wind conditions.	Fixed frontal area makes it prone to higher aerodynamic loads in strong winds, affecting stability and efficiency.
Maneuverability	High dynamic responsiveness enables complex flight maneuvers such as sharp turns and rolls.	Limited maneuverability due to fixed wing structure, making it less capable of performing complex flight maneuvers.
Reliability	Involves complex mechanical folding structures, which may require high material precision and manufacturing accuracy, potentially reducing reliability and increasing maintenance complexity.	Simple design with low mechanical failure probability, offering high reliability and ease of manufacturing and maintenance.
Energy efficiency	Dynamic wing adjustment reduces flight drag, optimizing energy use, particularly for long-duration missions or high-load scenarios.	Fixed wing span and aerodynamic characteristics prevent dynamic drag and energy optimization, leading to lower efficiency during prolonged missions.

**Table 2:** Estimated parameters.

Parameter Name	Parameter Size (mm)
Bird body (including tail)	410
Single-wingspan	275
Inner wingspan	195
Outer wing length	80
Mean geometric chord length of the wings	45

**Table 3:** Basic configuration parameters of the virtual wind tunnel [24,25,26].

Condition Name	Condition Description	Condition Parameters
Boundary region	Virtual aerodynamics	Rectangular prism 4 m × 2.5 m × 3 m
	Air	$\mathrm{Density}\;\rho =1.200\;{\mathrm{kg}/m}^{3}$ Temperature T=300.15 K $\mathrm{Dynamic}\;\mathrm{viscosity}\;\mathrm{coefficient}\;\mu =1.789\times 10^{-5}\;\mathrm{Pa}\cdot s$
Fluid		
Inlet	Incoming flow velocity	V=4 m/s
Outlet	Pressure boundary	$P_{out}=0\;pa$
Fuselage model	Along the positive direction of the *X*-axis	Initial angle of attack 0° Velocity along each axis is V=0 m/s

**Table 4:** Average aerodynamic characteristics at different frequencies.

Flapping Frequency	Lift Coefficient	Drag Coefficient	Lift-to-Drag Ratio
2	0.2149	0.0268	8.0187
4	0.153	0.0289	5.2941
5	0.4523	0.0167	27.084
6	0.0971	0.0270	3.5963
8	0.1232	0.0276	4.4638

**Table 5:** Average aerodynamic characteristics at different speeds.

Flapping Speed	Lift Coefficient	Drag Coefficient	Lift-to-Drag Ratio
2	0.0172	0.0090	1.9125
3	0.0707	0.0177	3.9961
4	0.1530	0.0297	5.1487
5	0.2811	0.0437	6.4361
6	0.4444	0.0593	7.4891

## Data Availability

Data is contained within the article.
